# MicroRNAs in anthracycline cardiotoxicity: biomarkers, mechanisms, and therapeutic advances

**DOI:** 10.3389/fcvm.2025.1614878

**Published:** 2025-08-22

**Authors:** Hongyun Mao, Jing Hu, Chenshuo Yu, Sicong Xie, Cheng Chang, Juanjuan Peng, Yang Zhang

**Affiliations:** ^1^ Department of Rehabilitation Medicine, School of Acupuncture-Moxibustion and Tuina and School of Health Preservation and Rehabilitation, Nanjing University of Chinese Medicine, Nanjing, China; ^2^ School of Pharmacy, Nanjing University of Chinese Medicine, Nanjing, China; ^3^ Department of Neurosurgery, NanJing JiangNing Hospital of Chinese Medicine and Affiliated Jiangning Hospital of Chinese Medicine, China Pharmaceutical University, Nanjing, China; ^4^ Department of Cardiology, Kunshan Hospital of Traditional Chinese Medicine, Kunshan, China

**Keywords:** anthracycline, cardiotoxicity, microRNAs, biomarker, mechanisms

## Abstract

**Background:**

Anthracycline-based chemotherapy is a highly effective treatment for numerous cancers, yet its clinical use is severely limited by cumulative, dose-dependent cardiotoxicity. MicroRNAs (miRNAs), as key post-transcriptional regulators of gene expression, play a pivotal role in the pathophysiology of cardiovascular disease, but their specific functions in anthracycline-induced cardiotoxicity (AIC) require systematic elucidation.

**Purpose:**

This review aims to systematically summarize current research on the key miRNAs, their molecular targets, and associated signaling pathways that regulate AIC, while also exploring their potential as biomarkers for early diagnosis and as therapeutic targets for intervention.

**Methods:**

A comprehensive literature search was conducted in PubMed, Web of Science, and Scopus databases for relevant studies published up to April 2025. Search terms included combinations of “microRNA,” “anthracycline,” “doxorubicin,” “cardiotoxicity,” and “cardiomyopathy.”

**Results:**

A complex network of miRNAs is involved in the regulation of AIC. Pro-toxic miRNAs, such as miR-34a and miR-146a, exacerbate cardiomyocyte apoptosis and oxidative stress by targeting Sirtuin 1 (SIRT1) and anti-apoptotic proteins. In contrast, cardioprotective miRNAs, such as miR-21 and miR-133a, mitigate cardiac injury by inhibiting fibrosis and apoptosis pathways. This network dynamically influences the onset and progression of AIC, affecting key processes including oxidative stress, autophagy, fibrosis, and apoptosis.

**Conclusion:**

MiRNAs play a dual role in the pathomechanisms of AIC, acting as both pathogenic factors and protective agents. A deeper understanding of this regulatory network provides a solid theoretical foundation for developing novel miRNA-based diagnostic biomarkers and intervention strategies to manage AIC. Future research should focus on validating clinical biomarker panels and optimizing targeted delivery systems.

## 1 Introduction

Malignant tumors represent one of the foremost threats to human health globally, with a mortality rate second only to cardiovascular diseases (1). As potent chemotherapeutic agents, anthracyclines have significantly improved prognoses for a variety of cancers, including breast cancer, leukemia, and lymphoma (2). Since the advent of daunorubicin (DNR), various anthracyclines such as doxorubicin (DOX), epirubicin (EPI), and idarubicin (IDA) have achieved tremendous success in oncology (3–8). Their antitumor effects are primarily achieved by intercalating into DNA, inhibiting topoisomerases, and generating reactive oxygen species (ROS), which lead to DNA and protein damage (2).

However, despite their remarkable efficacy, the cumulative use of anthracyclines is closely associated with dose-dependent cardiotoxicity, which can manifest as cardiomyopathy, arrhythmias, and even heart failure (9, 10). Clinically, anthracycline-induced cardiotoxicity can affect patients of all ages with varying severity (11, 12). Clinical investigations show that cancer patients treated with anthracyclines have a nearly tenfold higher risk of developing heart failure compared to healthy individuals (2, 13, 14). Although dexrazoxane (DRZ) is the only cardioprotective agent approved by the U.S. Food and Drug Administration (FDA), it has limitations, including potential interference with the anticancer activity of anthracyclines and an increased risk of neutropenia and thrombocytopenia (15–17).

**Figure 1:**
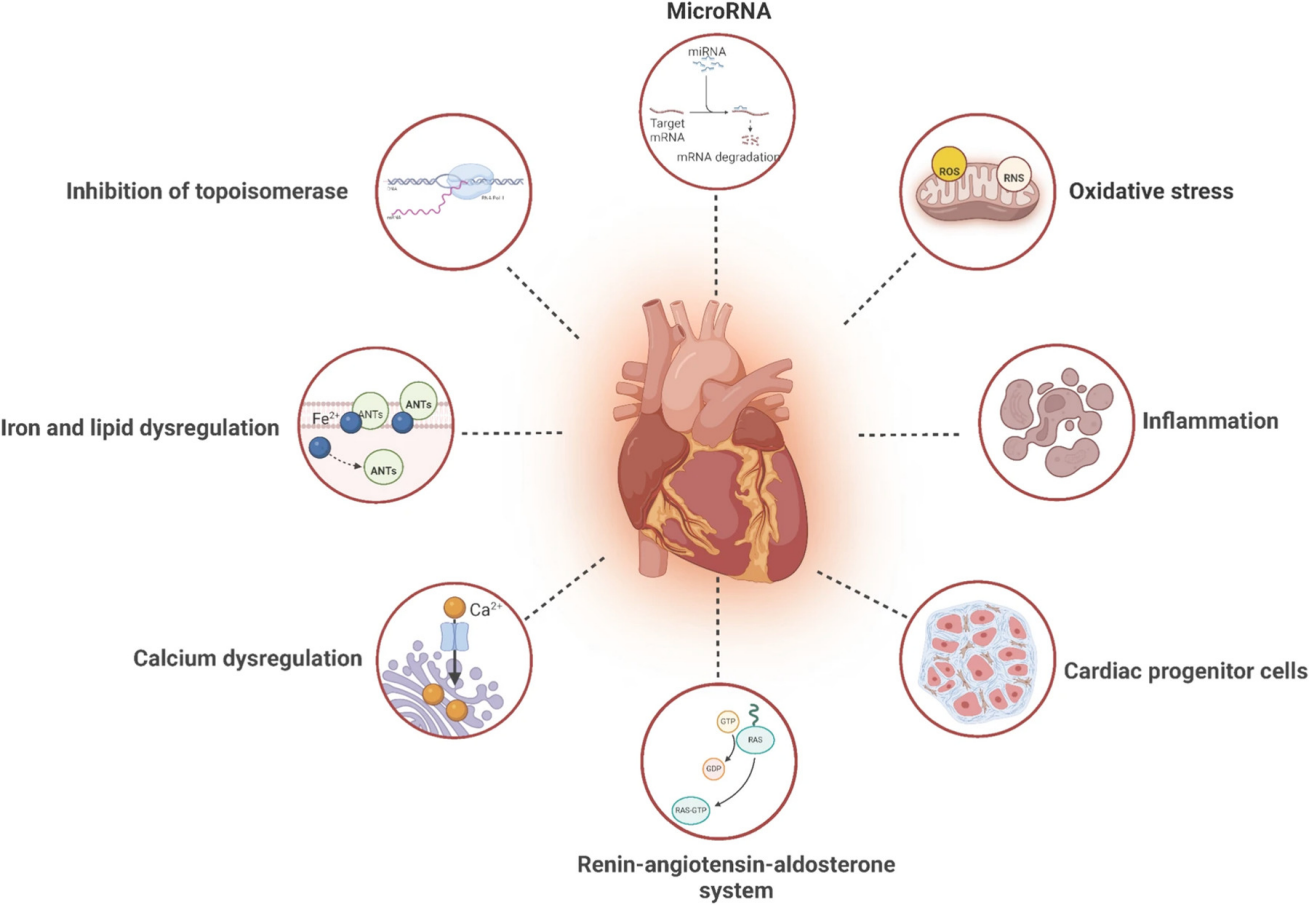
Mechanisms of anthracycline-induced cardiotoxicity. The mechanism of anthracycline-induced cardiotoxicity is believed to involve the inhibition of TOP 2β, iron ion metabolism, mitochondrialdysfunction, myocardial fiber dissolution, sarcoplasmic reticulumcalcium imbalance, oxidative stress, cell apoptosis and miRNAs. Reproduced with permission from “Mechanisms of AIC with pharmacologic targets” by Ziyu Kuang, Yuansha Ge, Luchang Cao, Xinmiao Wang, Kexin Liu, Jiaxi Wang, Xiaojuan Zhu, Min Wu and Jie Li, licensed under CC BY 4.0.

The primary mechanisms of anthracycline cardiotoxicity involve the inhibition of topoisomerase IIβ (TOP2β), dysregulation of iron metabolism, mitochondrial dysfunction, myocardial fibrosis, sarcoplasmic reticulum calcium imbalance, oxidative stress, apoptosis, and aberrant microRNA (miRNA) expression (18) (Figure 1). In recent years, advances in molecular cardiology have revealed the critical role of non-coding RNAs, particularly miRNAs, in mediating these pathophysiological processes (19–21).

MiRNAs are small non-coding RNA molecules, approximately 19–25 nucleotides in length, that play a vital role in regulating gene expression post-transcriptionally. Since their discovery in the early 1990s (22), over 2,000 miRNAs have been identified in humans, regulating more than 30% of human genes (23). MiRNAs have been confirmed to be involved in a wide range of physiological and pathological processes, including development, differentiation, apoptosis, and disease progression (24). Given their stability in bodily fluids and tissue specificity, miRNAs have emerged as highly promising biomarkers for cardiovascular diseases, including drug-induced cardiotoxicity. The latest authoritative reviews indicate that the field is transitioning from a “discovery” phase to the threshold of “clinical validation,” with a major focus now on constructing multi-molecule biomarker panels to enhance diagnostic precision (25, 26). This review aims to systematically summarize the current evidence on miRNA regulation in anthracycline cardiotoxicity and to explore their potential as diagnostic biomarkers and therapeutic targets.

## 2 Literature search methodology

We conducted a comprehensive literature search in PubMed, Web of Science, and Scopus databases to identify relevant studies published up to April 2025. Search terms included combinations of “microRNA” or “miRNA” with “anthracycline,” “doxorubicin,” “daunorubicin,” or “epirubicin,” and “cardiotoxicity,” “cardiomyopathy,” or “heart failure.” We prioritized original research articles, systematic reviews, and meta-analyses, while excluding case reports and non-English publications. Studies were selected based on their relevance to miRNA expression in cardiac tissue or circulation following anthracycline exposure, with a particular emphasis on mechanistic insights, biomarker potential, and therapeutic applications.

## 3 Fundamentals of miRNAs in cardiac biology

### 3.1 miRNA biogenesis and function

MiRNAs originate from genes and are transcribed by RNA polymerase II into primary miRNAs (pri-miRNAs), which are then processed in the nucleus by the microprocessor complex into precursor miRNAs (pre-miRNAs) (27). These pre-miRNAs are transported to the cytoplasm by exportin-5, where they are cleaved by the endonuclease DICER to form double-stranded miRNAs (28). One strand is integrated into the RNA-induced silencing complex (RISC), while the other is degraded. The mature miRNA guides the RISC to target mRNAs, typically by binding to the 3′-untranslated region, leading to translational repression or mRNA degradation (19).

In the cardiovascular system, miRNAs regulate cardiac development, function, and stress responses by orchestrating fundamental processes such as cardiomyocyte proliferation, apoptosis, fibrosis, hypertrophy, and metabolism (24). For instance, the muscle-specific miRNAs miR-1 and miR-133a are central to cardiac health. Mechanistically, they attenuate pathological hypertrophy by inhibiting the calcium-calcineurin-Nuclear Factor of Activated T-cells (NFAT) signaling pathway (29) and modulate cell survival by targeting apoptosis-related genes (30). Specifically, miR-133a enhances cardiomyocyte survival by repressing genes like Apaf-1 and caspases-3/9 (31). Conversely, other miRNAs, such as miR-155, promote pathological remodeling by activating inflammatory pathways (32). Furthermore, miR-34a facilitates cardiomyocyte apoptosis and senescence by inhibiting SIRT1, highlighting the deep involvement of miRNAs in cellular stress responses (33, 34). Dysregulation of these cardiac miRNAs is a hallmark of numerous cardiovascular pathologies (29).

### 3.2 Tissue versus circulating miRNAs: from local pathology to systemic biomarkers

In the context of anthracycline cardiotoxicity, miRNA expression profiles exhibit significant spatial heterogeneity. Differentiating between *in situ* expression changes within cardiac tissue and fluctuating levels in the peripheral circulation is crucial for understanding the underlying pathophysiology and for discovering clinically relevant biomarkers. Tissue-level miRNAs directly reflect the immediate molecular regulatory events within cardiomyocytes under drug-induced stress, whereas circulating miRNAs represent the systemic release of damage signals, collectively offering complementary diagnostic perspectives.

Table 1 provides a comprehensive summary of key dysregulated miRNAs across different models. At the cardiac tissue level, the dysregulation of specific miRNAs is directly linked to the core mechanisms of cardiotoxicity. For instance, in a rat model, cumulative DOX dosage led to a dose-dependent downregulation of let-7g and upregulation of miR-208b in myocardial tissue, changes associated with apoptosis and mitochondrial dysfunction (45, 46). Similarly, the significant upregulation of miR-34a in mouse cardiac tissue has been confirmed as a key pro-apoptotic signal (36). These tissue-level miRNAs, including miR-23a and miR-205 (47, 48), constitute the direct molecular blueprint of the heart's response to anthracycline toxicity. Although their detection relies on invasive sampling, they provide irreplaceable value for mechanistic studies.

**Table 1:** Dysregulated miRNAs in different models and clinical conditions.

miRNA	Model/Patient Condition	Sample	Change	Key Finding Summary	References
miR-1298-5p	Rabbit model (chronic daunorubicin)	Cardiac tissue	↑ (Upregulated)	Showed the strongest upregulation (29-fold) at the early subclinical stage (5 weeks).	Adamcova et al. (35)
miR-34a-5p	Rabbit model (chronic daunorubicin)	Cardiac tissue and Plasma	↑ (Upregulated)	Upregulation increased from 6.2-fold at 5 weeks to 76-fold at 10 weeks; plasma levels correlated with toxicity severity.	Adamcova et al. (35)
miR-34a	Mouse model (doxorubicin)	Cardiac tissue	↑ (Upregulated)	Exhibited strong dose-dependent upregulation; a key pro-apoptotic miRNA.	Desai et al. (36)
miR-146a	Mouse model (acute doxorubicin)	Cardiac tissue	↑ (Upregulated)	Upregulated during acute toxicity, targets ErbB4, and promotes cardiomyocyte apoptosis.	Horie et al. (37)
miR-1	Breast cancer patients (doxorubicin)	Plasma	↑ (Upregulated)	Identified as an early marker of cardiac injury, preceding troponin elevation.	Rigaud et al. (38)
	Rat model (coronary artery ligation)	Serum	↑ (Upregulated)	Levels increased 200-fold, indicating rapid release after myocardial injury.	Cheng et al. (39)
miR-133a	Acute myocardial infarction patients	Serum	↑ (Upregulated)	Showed a faster rise compared to troponin, indicating high sensitivity.	Kuwabara et al. (40)
	Doxorubicin-induced cardiac injury	Plasma	↑ (Upregulated)	Elevated plasma levels support its role as a diagnostic indicator.	Yu et al. (31)
miR-208a/b	Rat models & Cancer patients	Plasma	↑ (Upregulated)	Heart-specific miRNAs elevated in plasma, appearing earlier than traditional markers.	Creemers et al. (41)
miR-208	Rat model (high-dose doxorubicin)	Cardiac tissue	↓ (Downregulated)	Significantly downregulated in cardiac tissue at a high dose (15 mg/kg).	Doka et al. (42)
miR-499	Pediatric cancer patients	Plasma	↑ (Upregulated)	Elevated plasma levels detected after anthracycline treatment.	Wang et al. (43)
miR-499a-5p	Mouse model (doxorubicin)	Cardiac tissue & Plasma	↓ (Tissue), ↑ (Plasma)	Decreased tissue levels with increased plasma levels, indicating utility as a circulating biomarker of injury.	Ma et al. (44)
miR-29b	Pediatric cancer patients	Plasma	↑ (Upregulated)	Elevated plasma levels detected after anthracycline treatment.	Wang et al. (43)
let-7g	Rat model (chronic doxorubicin)	Cardiac tissue	↓ (Downregulated)	Dose-dependent downregulation linked to apoptosis and mitochondrial dysfunction.	Fu et al. (45) and Vacchi-Suzzi et al. (46)
miR-205	NMRI mice (doxorubicin)	Cardiac tissue	↓ (Downregulated)	Significantly downregulated in cardiac tissue, associated with DOX-induced injury.	Hanousková et al. (47)
miR-23a	Rat model (doxorubicin)	Cardiomyocytes	↑ (Upregulated)	Linked to mitochondrial dysfunction via the PGC-1*α*/DRP1 pathway.	Du et al. (48)
miR-21	Mouse model (doxorubicin)	Cardiomyocytes	↑ (Upregulated)	Overexpression reduces apoptosis by targeting BTG2; has anti-apoptotic properties.	Tong et al. (49)
miR-30 family	Rat model (doxorubicin)	Cardiomyocytes	↓ (Downregulated)	Exerts cardioprotective effects by regulating β-adrenergic signaling and reducing apoptosis.	Roca-Alonso et al. (50)
miR-320a	Mouse model (doxorubicin)	Cardiac tissue	↑ (Upregulated)	Exacerbates apoptosis by targeting VEGF-A.	Yin et al. (51)
Multiple	hPSC-derived cardiomyocytes (doxorubicin)	Cell culture	Dynamic changes	miR-34a/b, miR-187, miR-199a/b, miR-146a, etc., showed dynamic changes.	Holmgren et al. (52)

In contrast, circulating miRNAs hold immense clinical translation potential due to the feasibility of non-invasive detection. Studies have shown that some miRNAs highly expressed in the heart, such as miR-1 and miR-133a, are rapidly released into the peripheral blood following myocardial injury (38–40). In breast cancer patients treated with doxorubicin, an increase in plasma miR-1 levels even preceded the traditional biomarker troponin, demonstrating its superiority as an early warning indicator (38). Furthermore, the elevation of heart-specific or -enriched miRNAs like miR-208a/b and miR-499 in patient plasma further solidifies their status as “liquid biopsy” markers for cardiac damage (41, 43).

Crucially, some miRNAs show significant changes in both tissue and circulation, bridging the gap from local pathology to systemic biomarkers. MiR-34a-5p is a prime example: in a chronic cardiotoxicity rabbit model, its expression in myocardial tissue increased 6.2-fold at 5 weeks to 76-fold at 10 weeks, while its plasma levels also correlated with the severity of toxicity (35). Another elegant example is miR-499a-5p, which was downregulated in doxorubicin-treated cardiac tissue but significantly elevated in plasma (44). This inverse pattern strongly suggests its passive release from damaged cardiomyocytes, providing direct evidence for interpreting the origin of circulating miRNAs. Therefore, an integrated analysis of tissue and circulating miRNA profiles can not only elucidate the deep mechanisms of cardiotoxicity but also help screen for high-value biomarkers that are both pathologically relevant and clinically accessible.

## 4 MiRNA dysregulation in anthracycline-induced cardiotoxicity

### 4.1 Temporal dynamics: capturing the molecular trajectory from early warning to late-stage damage

The progression of anthracycline cardiotoxicity is a continuum from subclinical dysfunction to overt heart failure, a trajectory precisely mirrored by the dynamic evolution of miRNA expression profiles. Different miRNAs peak at various stages of toxicity, revealing their specific roles in the initiation, progression, and exacerbation of cardiac injury (Table 2). *In vivo* models across multiple species consistently illustrate this temporal nature. A pioneering study using a rabbit model of chronic daunorubicin cardiomyopathy found that during the early, subclinical stage (5 weeks), miR-1298-5p was the most significantly upregulated molecule (29-fold). However, as the disease progressed to the late stage of overt cardiac dysfunction (10 weeks), the expression of miR-34a-5p soared to 76 times the baseline level, making it a hallmark of advanced damage (35). In an acute toxicity model, myocardial miR-146a was rapidly upregulated in mice following doxorubicin administration, mediating early cardiomyocyte apoptosis by inhibiting the ErbB4 signaling pathway (37).

**Table 2:** Temporal dynamics of miRNA expression.

miRNA	Model/Condition	Anthracycline and Dose	Time Point	Temporal Expression Pattern	References
miR-1298-5p, miR-34a-5p	Rabbit chronic cardiomyopathy model	Daunorubicin (3 mg/kg)	5 weeks (early/subclinical) & 10 weeks (late/overt)	miR-1298-5p is most upregulated at 5 weeks. miR-34a-5p is modestly upregulated at 5 weeks but becomes the most significant change at 10 weeks (76-fold).	Adamcova et al. (35)
miR-214, miR-424, etc.	Human pluripotent stem cell-derived cardiomyocytes (hPSC-CMs)	Doxorubicin (unspecified)	Acute and chronic phases	miR-214 and miR-424 are differentially expressed at different time points, highlighting roles in different stages of cardiotoxicity.	Holmgren et al. (52)
miR-146a	Mouse cardiac injury model	Doxorubicin (20 mg/g)	Acute toxicity phase	Significantly upregulated during the acute phase, promoting early cardiomyocyte apoptosis.	Horie et al. (37)
miR-27a-5p, miR-99b-5p, miR-133a, miR-181a-5p, and miR-34a-5p	Isolated ventricular myocytes	Doxorubicin (10 µM)	Hours (early) and long-term exposure (late)	Revealed distinct “early-response miRNAs” (appearing within hours) and “late-response miRNAs” (after long-term exposure).	Domínguez Romero et al. (53)

*In vitro* studies further validate these time-dependent changes. In human pluripotent stem cell-derived cardiomyocytes (hPSC-CMs), DOX exposure triggered dynamic changes in multiple miRNAs, including miR-34a, miR-199a, and miR-146a. Notably, miR-214 and miR-424 showed differential expression at different time points, suggesting their respective involvement in the acute and chronic phases of cardiotoxicity (52). Studies on isolated ventricular myocytes have also confirmed the existence of “early-response miRNAs” (appearing within hours) and “late-response miRNAs” (appearing after long-term exposure) (53). Crucially, the appearance of certain circulating miRNAs may surpass that of traditional clinical biomarkers. For instance, a clinical study found that the elevation of plasma miR-1 preceded the increase in cardiac troponin I (cTnI) and showed superior discriminatory power in distinguishing patients with and without cardiotoxicity (38). Similarly, in patients with acute myocardial infarction, miR-133a rose faster than troponin (40). These findings highlight the immense potential of miRNAs as early, highly sensitive biomarkers, offering a window for intervention before irreversible myocardial damage occurs.

### 4.2 Dose-dependence: miRNAs as molecular dosimeters for cumulative toxicity

The expression of specific miRNAs in cardiac tissues varies significantly depending on the dose of anthracyclines administered. In a rat model of DOX-induced cardiotoxicity, animals treated with increasing cumulative doses of DOX (6 mg/kg, 12 mg/kg, and 18 mg/kg) showed a dose-dependent downregulation of miR-let-7g, alongside the upregulation of miR-208b, miR-216b, miR-215, miR-34c, and miR-367. These miRNAs were associated with apoptosis, oxidative stress, and mitochondrial dysfunction (45, 46).

The cumulative dose of anthracyclines is the primary determinant of cardiotoxicity, and a tight dose-response relationship exists between drug exposure and miRNA expression levels. This positions miRNAs as potential “molecular dosimeters” for quantifying the extent of cumulative cardiac damage (Table 3). In a chronic DOX-treated rat model, as the cumulative dose increased from 6 mg/kg to 18 mg/kg, myocardial let-7g expression showed a dose-dependent downregulation, while several other miRNAs, including miR-208b and miR-216b, were dose-dependently upregulated (45, 46). Another mouse study found that once the cumulative DOX dose exceeded 18 mg/kg, the expression of 21 miRNAs, including miR-34a, changed significantly, demonstrating a clear toxicity threshold effect (36). This threshold phenomenon was confirmed in another study where a high dose (15 mg/kg) of DOX led to a significant downregulation of miR-208 and miR-499 in rat myocardium, while a low dose (3 mg/kg) had no significant effect (42).

**Table 3:** Dose-Dependent miRNA regulation.

miRNA	Model	Anthracycline and Dose	Dose-Dependent Finding	References
let-7g, miR-208b, miR-216b, miR-215, miR-34c, miR-367	Rats	DOX (cumulative doses of 6, 12, 18 mg/kg)	let-7g showed dose-dependent downregulation, while the other listed miRNAs were dose-dependently upregulated.	Fu et al. (45) and Vacchi-Suzzi et al. (46)
miR-34a (and 20 others)	Male B6C3F1 mice	DOX (cumulative dose >18 mg/kg)	Expression of 21 miRNAs was significantly altered. miR-34a showed strong dose-dependent upregulation.	Desai et al. (36)
miR-34a, miR-205	NMRI mice	DOX (maximum recommended dose)	The maximum dose led to significant upregulation of miR-34a and downregulation of miR-205.	Hanousková et al. (47)
miR-208, miR-499, miR-1, miR-133a	Rats	DOX (high dose: 15 mg/kg vs. low dose: 3 mg/kg)	miRNAs were significantly downregulated at the high dose, with no significant changes detected at the low dose, suggesting a threshold effect.	Doka et al. (42)
miR-208a, let-7g	Rats	Standard DOX vs. Liposomal DOX	Liposomal DOX caused smaller changes in miRNAs compared to standard DOX, corresponding to lower cardiotoxicity.	Novak et al. (54)

Furthermore, drug formulation also influences miRNA expression profiles, indirectly reflecting a dose effect. Under the same dosage regimen, liposomal DOX, as a dosage form designed to reduce heart exposure, resulted in a significant decrease in expression of miR-208a and let-7g in rat hearts compared with standard doxorubicin, consistent with the lower cardiotoxicity of liposomal preparations (45). Although inconsistent reports exist regarding the expression trends of specific miRNAs, which may stem from differences in species, administration protocols, or detection time points, the core principle of “dose-dependence” is generally applicable. *In vitro* models also support this conclusion, showing that increasing DOX concentrations in cultured cardiomyocytes triggers graded changes in miRNA expression levels (52, 53). This precise dose-response relationship provides a powerful molecular tool for preclinical evaluation of the cardiac safety of different drugs or formulations, as well as for clinical monitoring of individualized cumulative toxicity risk.

### 4.3 Biomarkers: a critical assessment of integrated potential and practical challenges

The pathophysiology of anthracycline cardiotoxicity is intricate, involving interconnected pathways such as oxidative stress, DNA damage, mitochondrial dysfunction, inflammation, and apoptosis. MiRNAs are key nodes in the regulatory network governing these processes. Therefore, the dysregulation of specific miRNAs is not only a consequence of cardiac injury but may also be a driver of pathological progression. This dual role endows them with great value as biomarkers. However, translating these findings into reliable clinical tools requires a prudent assessment of their integrated potential and inherent limitations.

An ideal biomarker should be detectable at the subclinical stage, reflect cumulative dose effects, correlate with long-term prognosis, and possess tissue specificity. Currently, no single miRNA meets all these criteria, but a well-designed miRNA panel shows great promise. Cardiac-enriched miRNAs such as miR-1, miR-133a, miR-208a/b, and miR-499 have drawn significant attention. Following injury, they are released from necrotic or apoptotic cardiomyocytes into the blood, leading to a rapid increase in circulating levels. The rise of miR-1 and miR-133a can outpace that of troponin, highlighting their potential as early injury markers (38, 40). MiR-208 and miR-499 are highly heart-specific, theoretically providing a more precise signal of cardiac damage (41), but their elevation typically reflects established cell death and may lack sensitivity in capturing subtle functional changes.

Unlike passively released biomarkers, stress and apoptosis-related miRNAs like miR-34a and miR-146a actively participate in pathological processes. MiR-34a serves as a classic example, directly driving cellular senescence and apoptosis by inhibiting targets like SIRT1 (33, 34). Its levels in both tissue and plasma increase significantly with worsening toxicity, especially in the late stages (35), making it not just a “marker” of injury but also a “driver” of disease progression. This gives miR-34a a dual role as an indicator of severity and a potential therapeutic target. However, its main challenge is the lack of specificity. MiR-34a is involved in aging and tumor suppression in multiple tissues, and its circuit level may be affected by cancer itself. Therefore, future research should shift from searching for a single biomarker to constructing a multi-dimensional miRNA panel. An ideal panel would integrate direct evidence of cardiomyocyte damage (e.g., miR-499), early stress signals (e.g., miR-146a), and molecules that reflect cumulative toxicity and prognosis (e.g., miR-34a). By algorithmically combining these, it may be possible to create a composite index with high sensitivity, specificity, and prognostic value.

## 5 Circulating miRNAs as clinical biomarkers

### 5.1 Critical evaluation of key candidate miRNAs and their diagnostic value

Circulating miRNAs have become a focal point of cardiotoxicity biomarker research due to their non-invasiveness, stability, and ability to reflect early pathological changes. Unlike “death markers” such as cTn, which only rise significantly after irreversible cardiomyocyte necrosis, miRNAs promise an earlier and broader diagnostic window. However, a critical and cautious approach must be taken when evaluating their clinical utility.

MiR-1 and miR-133a, as the two most abundant miRNAs in myocardium, exhibit rapidly elevated circulating levels following cardiac injury. Multiple studies confirm their appearance precedes cTn (38, 40). In patients treated with doxorubicin, miR-1 even showed superior discriminatory power in identifying cardiotoxicity compared to cTnI (38). While their early sensitivity is a significant advantage, these miRNAs are also highly expressed in skeletal muscle. Therefore, non-cardiac muscle damage (e.g., from chemotherapy side effects or strenuous exercise) could lead to false-positive results. Their clinical application must thus be interpreted in the context of the patient's overall clinical picture.

The miR-208 family and miR-499 are among the most promising candidates for addressing specificity issues as cardiac-specific (or enriched) miRNA families. They are expressed almost exclusively in the myocardium, so their elevation in circulation is considered direct evidence of cardiac injury (41, 43). The behavior of miR-499a-5p is particularly compelling; its downregulation in tissue occurs concurrently with its upregulation in plasma (44), providing strong support for the “injury-release” theory. Critical Analysis: However, their primary challenge is low abundance, requiring highly sensitive detection methods. Moreover, like the muscle-enriched miRNAs, they primarily reflect myocyte necrosis or severe damage and may lack sensitivity for capturing earlier, reversible functional changes.

MiR-34a-5p represents a unique candidate whose value extends beyond diagnosis to prognosis. In animal models, its plasma levels correlate closely with the severity and progression of cardiotoxicity, showing a dramatic multi-fold increase in the late stages (35). This suggests that miR-34a-5p could serve as a dynamic indicator of cumulative cardiac damage and functional decline. As previously mentioned, the broad biological roles of miR-34a (in aging, apoptosis, tumor suppression) make its specificity a major drawback. Large-scale clinical studies are needed to define a specific diagnostic threshold for its use in cancer patients.

Recent studies suggest that miR-409-3p may play a role in cardiotoxicity, particularly in the context of metabolic disorders. For example, in patients with type 2 diabetes and coronary heart disease, circulating miR-409-3p levels were associated with major adverse cardiovascular events (55). It exacerbates high glucose-induced apoptosis by targeting the CREB1/BCL2 pathway. This suggests that miR-409-3p may not be a specific marker of direct anthracycline toxicity, but rather a “risk-modifying” marker, reflecting the interplay between a patient's underlying metabolic state (like diabetes) and drug toxicity. Monitoring miR-409-3p could help identify ultra-high-risk individuals who are more susceptible to anthracyclines due to comorbid metabolic diseases.

### 5.2 From single markers to intelligent diagnostic models: comprehensive miRNA profiling analysis

As the limitations of single miRNAs become increasingly apparent, the research focus is shifting toward analyzing the complete circulating miRNA profile (miRNAome) using high-throughput sequencing and constructing multivariate diagnostic or predictive models. This strategy can integrate information from different pathological pathways, enhancing diagnostic robustness. Clinical studies have demonstrated its potential. For instance, a “signature” composed of multiple circulating miRNAs was significantly associated with treatment-related cardiac events (56), and another study identified 32 differentially expressed miRNAs pointing to key pathways like cell death and inflammation (57). A systematic review also concluded that combining heart-specific with inflammation-related miRNAs offers superior diagnostic performance (26). The key to future clinical application lies in developing standardized, clinically meaningful miRNA panels and validating their predictive value in large-scale, prospective clinical cohort studies.

### 5.3 Challenges and path to clinical translation

Despite their promising potential, the translation of miRNA biomarkers from research to routine clinical practice is contingent upon surmounting several formidable obstacles. The primary challenge lies in the pervasive lack of methodological standardization throughout the entire workflow, ranging from sample collection and processing, miRNA extraction, and quantification platforms (such as qPCR or NGS) to data normalization. This methodological heterogeneity severely hampers the ability to compare findings across studies, impeding the large-scale validation necessary for clinical implementation (19, 25). Beyond these technical hurdles lies the inherent biological complexity, as circulating miRNA levels can be influenced by a host of confounding factors, including age, sex, renal function, comorbidities, and even circadian rhythms. The influence of these variables must be systematically delineated to establish reliable reference ranges essential for accurate clinical decision-making. Currently, the most critical constraint is the insufficient level of clinical evidence, as most existing studies are small-scale retrospective analyses. Gaining regulatory approval and widespread adoption requires strong evidence from large, multi-center, prospective clinical trials to demonstrate not only clinical effectiveness but also the cost-benefit of miRNA-based diagnostics.

Despite their promising potential, the translation of miRNA biomarkers from research to routine clinical practice is contingent upon surmounting several formidable obstacles. The primary challenge lies in the pervasive lack of methodological standardization throughout the entire workflow, ranging from sample collection and processing, miRNA extraction, and quantification platforms to data normalization (19, 25). Beyond these technical hurdles lies the inherent biological complexity, as circulating miRNA levels can be influenced by a host of confounding factors, including age, sex, renal function, and comorbidities. Currently, the most critical constraint is the insufficient level of clinical evidence. Gaining regulatory approval and widespread adoption requires strong evidence from large, multi-center, prospective clinical trials to demonstrate both clinical effectiveness and cost-benefit.

## 6 Therapeutic applications of miRNAs: from target validation to precision intervention

MiRNAs are not only “messengers” of disease but also “regulators” of gene expression, making them highly attractive therapeutic targets. MiRNA therapies targeting the cardiotoxicity of anthracyclines mainly focus on inhibiting injurious miRNAs (using antagonists) or supplementing cardioprotective miRNAs (using mimics).

### 6.1 Inhibiting pro-apoptotic and pro-fibrotic miRNAs

One primary therapeutic avenue involves inhibiting pro-apoptotic and pro-fibrotic miRNAs whose upregulation drives cardiac injury. Targeting miR-34a, a central driver of cardiomyocyte apoptosis and senescence, has shown considerable promise, with inhibitors significantly reducing cell death and improving cardiac function in preclinical models (34). Similarly, silencing miR-208a can halt pathological remodeling and fibrosis (58), while inhibiting miR-320a counteracts its anti-angiogenic effects by restoring Vascular Endothelial Growth Factor A (VEGF-A) signaling, thereby improving myocardial perfusion (51). Other pro-toxic miRNAs include miR-21, which promotes fibrosis by activating the Transforming Growth Factor-β (TGF-β) pathway (32). However, this inhibitory strategy is fraught with challenges, principally the risk of off-target effects. Since many target miRNAs, including miR-34a, also function as vital tumor suppressors, systemic inhibition could paradoxically promote tumorigenesis. This underscores that the development of heart-specific delivery systems is a critical prerequisite for safe clinical translation.

### 6.2 Augmenting cardioprotective miRNAs

Complementing the inhibition of detrimental miRNAs, a parallel strategy focuses on restoring or augmenting the function of cardioprotective miRNAs that are suppressed during cardiotoxicity. This can be achieved by supplementing potent anti-apoptotic molecules like miR-21 (49) or members of the miR-30 family, which shield the heart from stress-induced damage by modulating β-adrenergic signaling (50). A recent landmark study exemplified the potential of this approach by using engineered exosomes to deliver miR-499a-5p directly to the heart, which significantly mitigated doxorubicin-induced cardiac dysfunction and established a new paradigm for targeted delivery (44). It is, however, crucial to maintain scientific rigor regarding the therapeutic goals. While enhancing miRNAs like miR-29b to reduce fibrosis is a valid objective (59), the notion of repairing established damage by promoting cardiomyocyte proliferation is currently unrealistic. Adult mammalian cardiomyocytes are terminally differentiated cells with exceedingly limited regenerative capacity, meaning the primary and most achievable goal of miRNA therapy is the protection of surviving myocytes and the inhibition of pathological remodeling, not cardiac regeneration.

A parallel strategy focuses on restoring or augmenting the function of cardioprotective miRNAs that are suppressed during cardiotoxicity. This can be achieved by supplementing potent anti-apoptotic molecules like miR-21 (which exhibits context-dependent dual roles) (49) or members of the miR-30 family, which shield the heart from stress-induced damage by modulating β-adrenergic signaling (50). Recent research has also highlighted the role of miRNAs in regulating autophagy, a cellular recycling process crucial for cardiomyocyte health. For instance, miR-223 protects myocardial tissue by inhibiting both apoptosis and autophagy via the AKT/mTOR pathway (60), while miR-145 induces protective autophagy by targeting Fibroblast growth factor receptor substrate 2 (FRS2) (61). A landmark study exemplified this approach by using engineered exosomes to deliver miR-499a-5p directly to the heart, which significantly mitigated DOX-induced cardiac dysfunction (44). While enhancing miRNAs like miR-29b to reduce fibrosis is a valid objective (59), the primary goal of miRNA therapy remains the protection of surviving myocytes and inhibition of pathological remodeling, rather than cardiac regeneration, given the limited regenerative capacity of adult mammalian cardiomyocytes.

### 6.3 Delivery systems: the key bottleneck to success

The successful clinical translation of miRNA-based therapeutics is critically dependent on overcoming the formidable bottleneck of drug delivery. The central challenge lies in transporting these unstable oligonucleotide drugs to target cardiac cells efficiently and safely. Significant technological progress is being made on several fronts. Targeted exosome delivery, using natural nanocarriers, has emerged as a highly promising strategy for achieving cardiac specificity (44). Synthetic platforms, including lipid and polymer-based nanoparticles, offer another viable path by encapsulating miRNA payloads to ensure stability and enable targeted delivery (62). While viral vectors like adeno-associated virus can provide long-term expression, their application is often limited by concerns over immunogenicity and integration risks. Furthermore, circular RNAs (circRNAs), which can function as endogenous miRNA “sponges” to inhibit specific miRNAs, offer an alternative mechanism for cardioprotection (21).

### 6.4 Natural compounds: a complementary strategy for indirect miRNA regulation

Several natural compounds, including resveratrol (63), paeoniflorin (64), and berberine (65), have been found to exert cardioprotective effects by modulating the expression of endogenous miRNAs. For example, paeoniflorin inhibits DOX-induced cardiomyocyte apoptosis by downregulating miR-1 expression (64). This strategy has the advantages of high safety and low cost, but its effects are indirect and its potency is limited, making it more suitable as a preventive or adjuvant therapy.

## 7 Future directions and challenges

Research on miRNAs in anthracycline cardiotoxicity has progressed from phenomenological discovery to in-depth mechanistic exploration and translational applications. To translate these basic research findings into tangible clinical benefits, a series of major challenges must be addressed.

### 7.1 Challenges and future directions for miRNA biomarkers

To elevate miRNA biomarkers to clinically indispensable tools, future research must pivot from discovery to rigorous validation. The foremost priority is the execution of large-scale, multi-center prospective cohort studies to correlate miRNA signatures with cardiac imaging and long-term clinical outcomes, thereby establishing definitive predictive panels. Simultaneously, it is necessary to adopt systems biology methods integrating multi-omics data to construct a comprehensive risk model that can elucidate the molecular basis of individual susceptibility. This macro-level validation must be complemented by micro-level mechanistic investigation, using advanced models like human cardiac organoids and CRISPR-based gene editing to dissect the precise regulatory networks of candidate miRNAs and establish a causal link between their dysregulation and pathophysiology.

### 7.2 Challenges and future directions for miRNA therapeutics

The therapeutic potential of miRNAs is contingent upon the development of a safe and efficient cardiac-specific delivery system. This delivery challenge is the primary bottleneck, as systemic administration of miRNA therapies risks significant off-target effects. Overcoming these technical and safety hurdles is intrinsically linked to the need for regulatory clarity and standardization. Establishing robust protocols for the production, quality control, and clinical evaluation of miRNA-based drugs is imperative for navigating the path to regulatory approval and ensuring that these innovative therapies can be safely and effectively deployed in the clinic.

## 8 Conclusion

After more than a decade of intensive research, microRNAs are poised to transition from discovery-phase molecules to clinically actionable tools in the management of anthracycline cardiotoxicity. The initial search for a single, definitive biomarker has evolved into a more sophisticated understanding: the greatest diagnostic power lies not in any individual miRNA, but in the integration of a multi-marker panel. Such a panel, combining myocyte-enriched markers of acute injury (miR-1, miR-499), stress-responsive indicators of cumulative damage (miR-34a), and heart-specific molecules (miR-208a), can create a composite signature with superior sensitivity and specificity for both early detection and prognostic stratification.

However, the path from this promising potential to routine clinical practice is obstructed by critical, yet addressable, challenges. The field must overcome the lack of methodological standardization and commit to large-scale, prospective clinical trials to validate these miRNA panels. For miRNA-based therapeutics, progress is fundamentally tethered to solving the challenge of targeted cardiac delivery to maximize efficacy and minimize off-target risks. By focusing on rigorous validation, technological innovation, and collaborative standardization, the scientific community can harness the power of miRNAs to create a new paradigm in cardio-oncology, ultimately protecting the hearts of cancer patients and improving both survival and quality of life.
